# Role of plasminogen activator inhibitor-1 (PAI-1) in age-related cardiovascular pathophysiology

**DOI:** 10.20517/jca.2024.38

**Published:** 2025-04-21

**Authors:** Alireza Khoddam, Douglas Vaughan, Lisa Wilsbacher

**Affiliations:** Feinberg Cardiovascular and Renal Research Institute, Feinberg School of Medicine Northwestern University, Chicago, IL 60611, USA.

**Keywords:** Cardiovascular disease, aging, senescence, vascular biology

## Abstract

Cardiovascular aging underpins the development of age-related diseases, including heart failure and vascular dysfunction, and is driven by molecular and cellular mechanisms described in the hallmarks of aging. Plasminogen activator inhibitor-1 (PAI-1), a key regulator of fibrinolysis, also mediates processes like vascular stiffness, cellular senescence, and immune evasion. This review highlights PAI-1’s role in cardiovascular aging with a special emphasis on senescence, a key hallmark of aging. It further explores PAI-1’s therapeutic potential, with a focus on its contribution to ECM remodeling, senescence signaling, and immune checkpoint regulation. Targeting PAI-1 could provide a promising strategy to mitigate age-related cardiovascular disease.

## Graphical Abstract

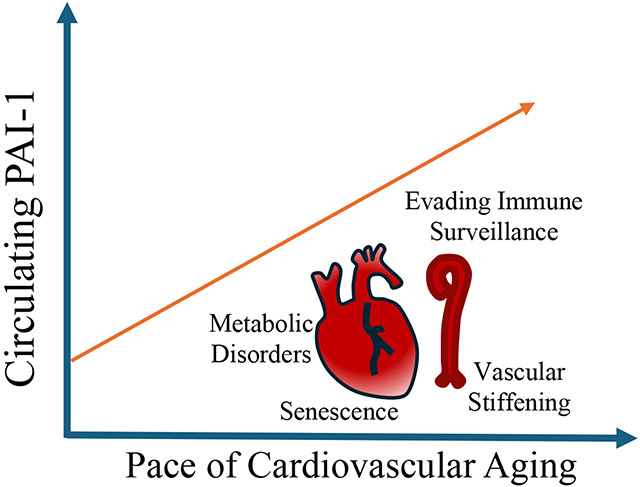

## INTRODUCTION

Cardiovascular disease (CVD) is the leading cause of death worldwide and is closely linked to aging^[1]^. As the global population over 60 is expected to double from 1 billion in 2020 to 2.1 billion by 2050^[2]^, addressing age-related cardiovascular conditions becomes increasingly urgent. The geroscience hypothesis posits that aging drives not only CVD but also other chronic diseases, suggesting that interventions targeting aging processes could mitigate these conditions^[3]^. Among these processes, the hallmarks of aging - such as genomic instability, cellular senescence, and disrupted proteostasis - provide a framework to dissect molecular and cellular changes that impair cardiovascular resilience, manifesting as hypertension, vascular stiffness, and heart failure^[4,5]^.

Plasminogen activator inhibitor-1 (PAI-1), a serine protease inhibitor, is closely linked to the hallmarks of aging and aging-related CVD^[6]^. PAI-1 was originally identified as the principal inhibitor of tissue-type plasminogen activator (t-PA) and urokinase-type plasminogen activator (u-PA), and PAI-1 thereby regulates fibrinolysis and extracellular matrix (ECM) remodeling^[7]^. Subsequent studies revealed additional t-PA targets that are impacted by PAI-1 inhibition and contribute to cardiovascular aging, such as fibroblast growth factor 23 (FGF23)^[8]^, brain-derived neurotrophic factor (BDNF)^[9,10]^, insulin-like growth factor binding protein 3 (IGFBP3)^[11]^, and matrix metalloproteinase-9 (MMP-9)^[12]^. PAI-1 is primarily secreted by endothelial cells, hepatocytes, and adipocytes, and it is cleared by the liver with a half-life of approximately 5 min^[13]^. A well-recognized pattern of regulation is its circadian variation, with levels peaking in the early morning^[14]^. Plasma levels of PAI-1 typically range from 5-20 ng/mL in healthy individuals, though this range increases with age^[15]^ and pathological states; in particular, hyperinsulinemia, obesity, and age lead to increased PAI-1 expression in adipose tissue^[16]^, and all of these conditions contribute to accelerated cardiovascular aging. In mice, transgenic overexpression of stabilized human PAI-1 drives an accelerated aging phenotype that includes alopecia, kidney fibrosis, amyloid deposition in the brain and liver, cardiac fibrosis, and spontaneous coronary artery thrombosis; these processes contribute to premature death^[17,18]^. Recent research indicates that PAI-1 exerts pleiotropic effects on physiology, making it a compelling target for combating age-related cardiovascular conditions. Its expression increases with age^[16,19]^ and is closely linked to cardiovascular aging through mechanisms like vascular stiffening, senescence, and immune modulation. This review focuses on these aspects - vascular stiffness, senescence, and immune evasion - because they represent critical nodes where PAI-1’s overexpression accelerates age-related pathology. We also explore why aging elevates PAI-1 levels, driven by factors such as oxidative stress, inflammation, and metabolic changes, and how this contributes to disease progression. By synthesizing these findings, we aim to highlight PAI-1’s therapeutic potential in mitigating cardiovascular aging.

## VASCULAR STIFFNESS

### Vascular stiffness overview

Vascular stiffness accelerates the development of CVD, especially in aging populations^[20]^. As arteries stiffen due to reduced elastin, increased collagen deposition, and calcification, they lose elasticity and become less responsive to changes in blood flow^[21]^. This pathophysiology leads to increased systolic blood pressure and pulse pressure, which raise left ventricular pressure; increased left ventricular pressure causes left ventricular hypertrophy that can progress to heart failure. Stiff arteries also fail to cushion the pulsatile flow of blood, damaging the microvasculature in organs like the brain, kidneys, and heart. Microvascular damage contributes to the development of hypertension and accelerates atherosclerosis, which together increase the risk of heart attacks and strokes^[22]^. At the physiological level, pulse wave velocity (PWV) is a key measure of arterial stiffness. PWV assesses the pulse wave transit time across a fixed distance, typically between the carotid and femoral arteries; the higher the velocity, the stiffer the vessel. PWV increases with age, particularly in the distal aorta^[23]^. Similarly, there is an associated increase in the diameter of the aorta with advancing age^[24]^.

At the molecular level, several pathways contribute to vascular stiffening. ECM composition changes with age, specifically with increased collagen deposition and elastin fiber degradation^[25]^. Furthermore, increased ECM stiffness due to increased collagen and decreased elastin was shown to promote transdifferentiation of fibroblasts to myofibroblasts^[26,27]^. Activated myofibroblasts secrete additional ECM components and exacerbate the stiffness of the vasculature^[28]^. Endothelial cells also modulate vascular tone and vasodilation through the release of nitric oxide (NO). Endothelial NO synthase (eNOS) produces NO, which diffuses to neighboring vascular smooth muscle cells (VSMCs). In VSMCs, NO activates soluble guanylate cyclase, increasing cyclic guanosine monophosphate (cGMP) levels, which in turn activate protein kinase G (PKG). PKG inhibits voltage-gated calcium channels, reducing calcium influx and leading to vasodilation^[29]^. NO production was also shown to negatively regulate VSMC collagen production^[30]^.

### PAI-1 contribution to vascular stiffness

**Figure 1.**
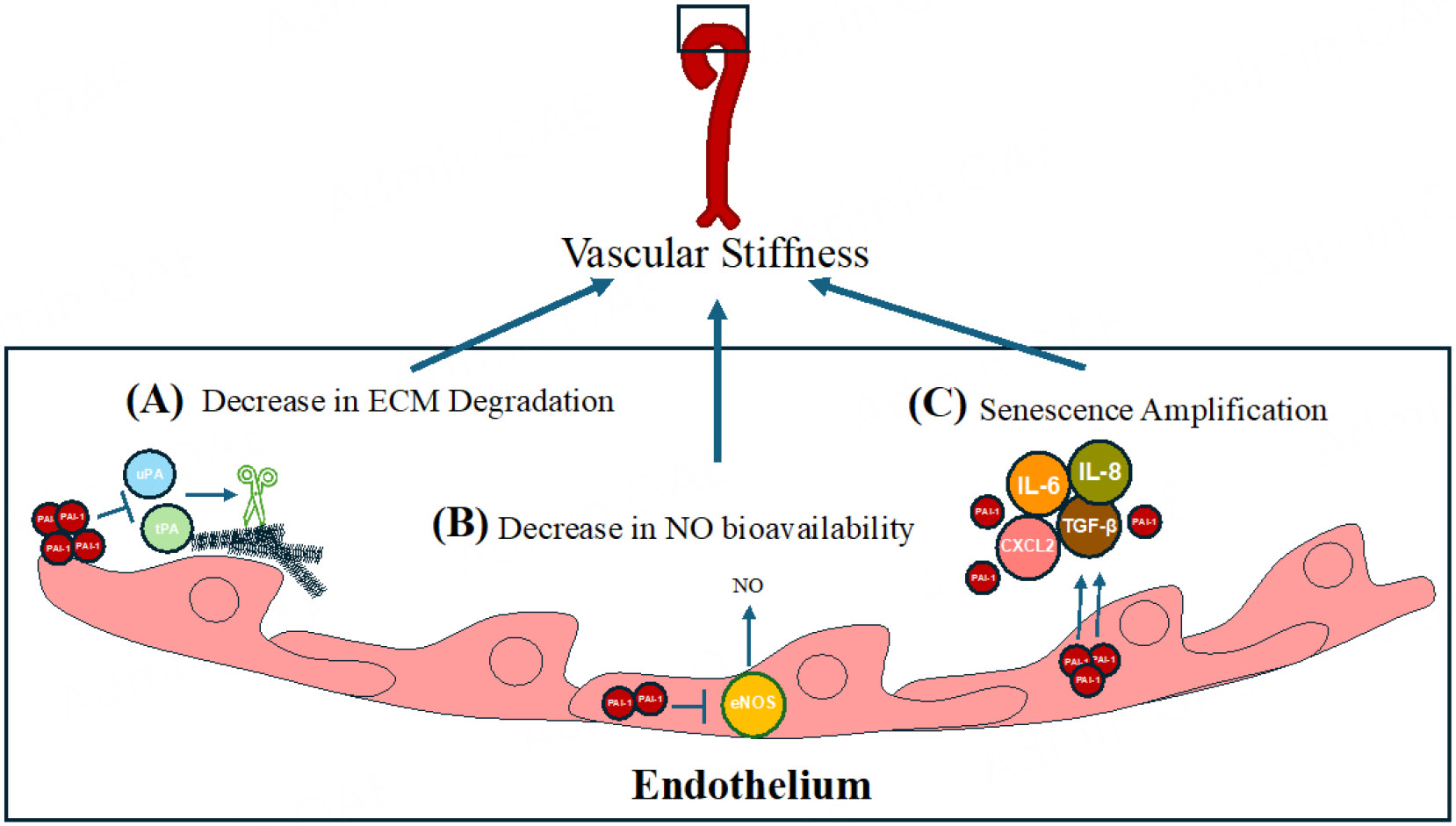
The role of PAI-1 in vascular stiffness and senescence amplification. (A) PAI-1 inhibits degradation of the ECM via inhibiting the tPA/uPA-mediated cleavage of metalloproteinases. (B) PAI-1 inhibits eNOS, reducing NO bioavailability and promoting vascular stiffness in the endothelium. (C) PAI-1 amplifies senescence by increasing SASP factors (IL-6, IL-8, TGF-β, CXCL2), which exacerbate vascular dysfunction and aging.

PAI-1 is a serine protease inhibitor (SERPIN), encoded by the *SERPINE1* gene, that was originally shown to reduce fibrinolysis through its inhibition of t-PA and u-PA^[7]^. Later work revealed that PAI-1 inhibition of t-PA and u-PA could contribute to ECM protein accumulation and increased vascular stiffness^[31]^. The uPA/tPA/plasmin/MMP proteolytic system regulates ECM turnover and homeostasis^[32]^. Since PAI-1 acts as an upstream inhibitor of uPA and tPA, it thus influences the tPA and uPA targets of plasmin and MMP activities, which degrade ECM components^[33,34]^. As a result, elevated PAI-1 levels are implicated in tissue fibrosis, driven by reduced ECM degradation^[35]^ and altered cellular migration^[36,37]^ [Figure 1A].

Subsequent studies identified novel molecular mechanisms by which PAI-1 might contribute to stiffness independently of its protease inhibitor activity. For example, L-NAME inhibits eNOS and increases blood pressure due to reduced NO availability; inhibition of PAI-1 was shown to blunt L-NAME-induced increase in blood pressure in mice^[38]^. Garcia *et al.* demonstrated that PAI-1 directly binds to eNOS, inhibits its activity in cultured endothelial cells, and that this interaction is ablated by using *SERPINE1* siRNA^[39]^; *in vivo* studies will determine whether this mechanism operates in the vasculature. Additionally, eNOS inhibition by PAI-1 might increase intracellular reactive oxygen species (ROS), potentially leading to cellular senescence (see below). This potential pathway is summarized in Figure 1B. Finally, recent atomic force microscopy studies in cultured VSMCs revealed that PAI-1 contributed to stiffness through increased Factin polymerization; pharmacological PAI-1 inhibition or *Serpine1* siRNA knockdown reduced intrinsic cellular stiffness, suggesting a mechanism independent of collagen and elastin content^[40]^. These studies suggest a model where increased PAI-1 decreases NO bioavailability and increases VSMC stiffness via Factin polymerization.

## SENESCENCE

### Senescence overview

Building on PAI-1’s role in vascular stiffness, its influence extends to cellular senescence, a hallmark of aging that further impairs vascular function and accelerates cardiovascular pathology. Specifically, senescent endothelial cells exhibit lower eNOS expression, linking PAI-1, NO bioavailability, and vascular aging^[41]^. Cellular senescence occurs in response to stressors like DNA damage, oxidative stress, and telomere shortening - all of which increase with age^[42]^. In the cardiovascular system, endothelial cells experience constant mechanical stress from blood flow, leading to high turnover, and a consequent telomeric shortening, which increases susceptibility to senescence^[43]^. The endothelium is vital for maintaining appropriate vascular tone, and the accumulation of senescent cells impairs vascular function^[44]^. For example, senescent cell accumulation in the aorta has been associated with pathologies such as aortic aneurysm^[45]^. At the molecular level, senescent cells upregulate the p53-p21 and/or RB-p16 pathways, blocking the cell cycle^[46]^. Although they do not proliferate, senescent cells remain metabolically active and secrete senescence-associated secretory phenotype (SASP) factors; besides PAI-1 itself, the SASP includes pro-inflammatory cytokines, growth factors, and proteases that induce senescence in neighboring cells. Senescent cells negatively affect the vasculature by impairing the regenerative capacity of endothelial and smooth muscle cells, disrupting tissue repair, and leading to ECM protein accumulation, all of which promote vascular stiffening and dysfunction^[47]^.

Current approaches to reducing senescent cell burden involve the induction of apoptosis of senescent cells (senolytic) or neutralizing SASP factors (senomorphic). For instance, targeting p16-positive cells for apoptosis extended median lifespan in mice by up to 25%^[48-50]^. Although these studies show promising results, the initial studies use genetically engineered mice with inducible caspase activation under the p16 promoter, posing challenges for direct clinical translation. The combination of dasatinib and quercetin (D + Q) effectively targets senescent cells with upregulated PI3K/Akt and BCL-2 pathways^[51]^. However, due to senescent cell heterogeneity, senolytics alone cannot eliminate all senescent cells^[52,53]^. Therefore, combining senolytic and senomorphic therapies that target both senescent cells and their amplification may enhance effectiveness. This approach emphasizes the need to identify and inhibit the most potent SASP factors.

### PAI-1 contribution to senescence

Multiple studies have demonstrated that PAI-1 directly contributes to senescence. Excess PAI-1 is associated with an increase in SASP factors such as IL-6, IL-1β, IL-8, CXCL2, and TGFβ^[54]^. Mechanistically, early reports showed that PAI-1 overexpression induced senescence in p53-deficient fibroblasts, indicating PAI-1 signals independently of p53^[55]^. Our group and others have previously reviewed PAI-1’s role in marking and inducing senescence^[54,56-58]^. Briefly, PAI-1 is synthesized and secreted by senescent cells, contributing directly to the development of cellular senescence downstream of p53 and upstream of insulin-like growth factor binding protein-3 (IGFBP-3). IGFBP-3 levels increase in senescent cells and directly stimulate senescence. t-PA mediates the proteolytic inactivation of IGFBP-3, while PAI-1 prevents this process, thereby promoting senescence.

Genetic PAI-1 deficiency and pharmacological PAI-1 inhibition reduce senescent cell markers *in vitro* and *in vivo*. In mice exposed to L-NAME, treatment with PAI-1 inhibitor TM5441 prevented increased senescence markers like p16 expression and telomere shortening in the aorta of L-NAME-treated mice^[38]^. In addition, PAI-1 inhibition in endothelial cells protected against homocysteine-induced senescence, preventing increased SA-β-Gal while enhancing NRF2 levels^[59]^. Similar protective effects against senescence were observed in murine cardiomyocytes exposed to doxorubicin^[60]^. Interestingly, treatment with D + Q lowers senescence markers p21 and BCL-xL but does not affect PAI-1 levels^[51]^, suggesting an opportunity to combine PAI-1 inhibitors with D + Q for more effective targeting of senescent cells.

Other than contributing to the aging phenotype, senescence plays a crucial role in tumor-protection mechanisms; hence, a concern with senolytic and senomorphic therapies is the uncertainty of long-term use^[46]^. Possible mechanisms underlying the alterations in senescent cells along the vasculature are summarized in Figure 1C. The involvement of PAI-1 in cancer leads to a broader perspective on how PAI-1 traverses the complex landscape of aging, intertwining with processes such as senescence, immune modulation, and cancer progression.

## EVADING IMMUNE SURVEILLANCE

Beyond its direct effects on stiffness and senescence, PAI-1 also modulates immune responses, linking its overexpression to immune evasion mechanisms that exacerbate vascular aging. Released senescence-associated secretory phenotype (SASP) factors, including pro-inflammatory cytokines such as IL-6, IL-1β, and TNF-α, recruit inflammatory cells to the vasculature, contributing to a low-grade inflammation termed “inflammaging”^[61]^. This chronic inflammation feeds into a cycle of cellular senescence, endothelial dysfunction, and arterial stiffening, accelerating pathologies like atherosclerosis that are central to cardiovascular aging^[44]^.

Recent studies have identified a connection between the immune checkpoint inhibitor Programmed Death Ligand-1 (PD-L1) and cellular senescence, with implications extending to both cardiovascular and cancer contexts. PD-L1, traditionally known for enabling cancer cells to evade immunosurveillance by binding to Programmed Cell Death Protein-1 (PD-1) on T cells and inducing T cell exhaustion^[62]^, is also upregulated in senescent fibroblasts following exposure to SASP factors via the JAK1/STAT3 pathway^[63]^. This upregulation allows senescent cells to evade clearance by CD8^+^ T cells, a mechanism paralleling immune evasion in tumors. Blocking the PD-1/PD-L1 interaction has been shown to reduce senescence markers *in vitro* and ameliorate senescence-associated phenotypes *in vivo*^[64-66]^. Similarly, the use of rapamycin suppressed the increased expression of PD-L1 in senescent cells; however, it did not affect other senescence markers like Lamin B1 and p16^[63]^. Furthermore, blocking the PD-1/PD-L1 interaction between senescent cells and CD8^+^ T cells was shown to reduce senescence markers *in vitro* and ameliorate senescence-associated phenotypes *in vivo*^[67]^. These reports suggest a possible mechanism where senescent cells directly upregulate PD-L1 to evade clearance by CD8^+^ T cells, akin to cancer cells. However, the role of PD-L1 in the cardiovascular system is complex, as immune checkpoint inhibitors targeting PD-1/PD-L1 are linked to increased atherosclerotic events by enhancing vascular inflammation^[68]^. This suggests a dual role for PD-L1, where its upregulation by PAI-1 may promote senescence-related immune evasion, yet its inhibition could exacerbate atherosclerosis. The interplay between PAI-1 and PD-L1 in this context remains underexplored and warrants further investigation.

PAI-1 appears to bridge these processes by upregulating PD-L1 expression in both tumor and senescent cells, mediated through the PAI-1/uPAR/LRP-1 complex and JAK1/STAT3 activation^[69]^. This upregulation may enable senescent cells in the vasculature to escape immune surveillance, contributing to the accumulation of dysfunctional cells that drive CV aging. Similarly, in cancer, PAI-1’s role in promoting metastasis - such as increasing ECM stiffness via integrinβ1-induced collagen I branching in endometrial tumor models^[70,71]^ - mirrors its effects on vascular stiffening in cardiovascular disease. The release of PAI-1 into the bloodstream with aging may thus create a pro-tumorigenic microenvironment while simultaneously exacerbating vascular inflammation and stiffness, linking cancer progression to cardiovascular pathology^[72-74]^.

The overlap between cancer and senescence is further evident in their shared secretory phenotypes, which release factors promoting inflammation and tissue remodeling^[73]^. While senescence is generally tumorsuppressive by halting proliferation, the accumulation of senescent cells in aged cardiovascular tissues can foster a pro-tumorigenic environment through SASP factors like PAI-1^[75]^. This duality suggests that age-related increases in circulating PAI-1 could amplify both cardiovascular dysfunction (atherosclerosis, hypertension) and cancer risk (metastasis), particularly in the context of vascular inflammation and ECM remodeling. For instance, PAI-1’s repression of adhesion genes via LRP-1 internalization may contribute to both senescence-associated tissue remodeling and cancer cell detachment during metastasis^[72]^, a process that could parallel vascular matrix changes in cardiovascular aging. Future research should elucidate the molecular mechanisms determining PAI-1’s context-specific effects, as well as explore whether PAI-1 inhibition could simultaneously mitigate vascular senescence and tumor progression. This dual-targeting potential could offer a novel therapeutic avenue for addressing the multisystem decline associated with aging, with particular relevance to cardiovascular disease prevention and management.

## PLEIOTROPIC ACTIONS OF PAI-1 AND OUTLOOK

While PAI-1 drives cardiovascular aging through stiffness, senescence, and immune modulation, its pleiotropic effects span additional physiological systems, broadening its therapeutic relevance.

### Metabolism and dyslipidemia

With increasing age, the risk and impact of dyslipidemia on cardiovascular health increase significantly. Aging is associated with changes in lipid metabolism, such as higher LDL cholesterol and triglycerides, and a decline in HDL cholesterol levels^[29]^. These lipid imbalances, combined with age-related changes in blood vessels - including reduced elasticity and increased arterial stiffness - create a “perfect storm” for atherosclerosis.

**Figure 2.**
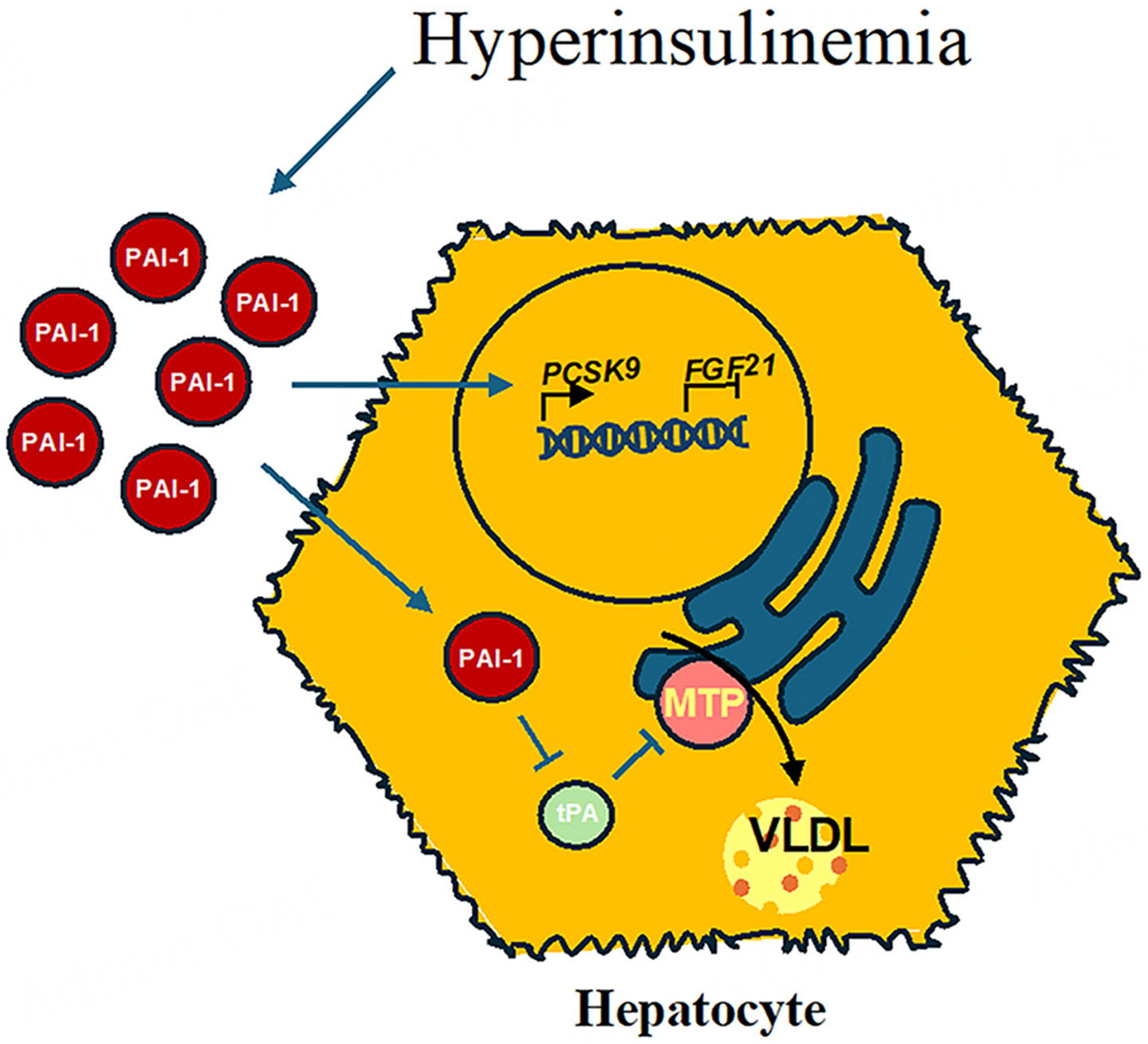
PAI-1’s role in dyslipidemia and metabolic dysfunction. Hyperinsulinemia elevates PAI-1 levels, which inhibit t-PA interaction with MTP, promoting VLDL assembly in hepatocytes. PAI-1 also upregulates PCSK9 and FGF21, exacerbating dyslipidemia and cardiovascular risk.

Notably, PAI-1’s role extends into the interplay between dyslipidemia and aging, exacerbating the risk of CVD^[76]^. Hyperinsulinemia, a hallmark of insulin resistance in aging and metabolic syndrome, is associated with amplified PAI-1 levels. Nordt *et al*. demonstrated that proinsulin and insulin induce PAI-1 expression in rabbits^[77]^. It has recently been reported that t-PA binds to microsomal triglyceride transfer protein (MTP) to prevent apolipoprotein B (ApoB) lipidation in hepatocytes^[78]^. Increased PAI-1 sequesters t-PA from MTP, promoting ApoB lipidation and very-low-density lipoprotein (VLDL) assembly^[78]^. This insulin-mediated PAI-1 activation exacerbates dyslipidemia by promoting VLDL assembly and impairs fibrinolysis, creating a feedback loop that accelerates atherosclerosis and cardiovascular risk. Additionally, treatment of mice with the PAI-1 inhibitor TM5614 was shown to reduce proprotein convertase subtilisin/kexin type 9 (PCSK9) expression while increasing fibroblast growth factor 21 (FGF21)^[79,80]^. High PCSK9 levels reduce LDL receptor availability, increasing blood cholesterol; therefore, PAI-1 inhibition might decrease circulating cholesterol by lowering PCSK9 levels. These mechanisms altering metabolism are summarized in Figure 2.

Currently, caloric restriction is the “gold standard” in preventing metabolic dysfunction^[81]^ and circulating PAI-1 levels are reduced following caloric restriction^[82]^. Specifically, during caloric restriction, liver-derived PAI-1 decreases^[83]^. This reduction in PAI-1 was associated with increased plasmin activity on muscle satellite stem cells and their expansion after injury^[83]^. Muscle atrophy during aging is linked to metabolic disorders due to impaired insulin sensitivity, reduced mitochondrial function, and chronic low-grade inflammation, which collectively disrupt protein synthesis and muscle regeneration^[84]^. These metabolic imbalances, exacerbated by elevated PAI-1 levels, suggest a mechanism by which PAI-1 accelerates the loss of muscle mass and strength, contributing to frailty and increased risk of metabolic diseases such as type 2 diabetes. Altogether, these findings add mechanistic insight into the association observed between PAI-1 and metabolic dysfunction pathologies which can accelerate cardiovascular aging.

### Renal function and Klotho link

In addition to metabolic impacts, PAI-1 influences renal function, intersecting with aging pathways like the FGF23-Klotho axis. The kidneys regulate blood pressure and vascular tone through the renin-angiotensin-aldosterone system^[85]^. Impaired renal function can cause fluid retention and hypertension, increasing cardiac workload and contributing to heart failure. Chronic kidney disease (CKD) disrupts normal renin-angiotensin-aldosterone processes, leading to hypertension and an increased risk of CVD such as atherosclerosis and left ventricular hypertrophy.

The FGF23-Klotho axis is important for kidney function and mineral homeostasis^[86]^. FGF23, secreted by osteocytes, regulates phosphate levels by inhibiting renal phosphate reabsorption and suppressing active vitamin D synthesis. Klotho acts as a co-receptor for FGF23, facilitating its interaction with fibroblast growth factor receptor 1 (FGFR1) in the kidney. Klotho hypomorphs result from a spontaneous insertional mutation that disrupts the promoter region of the mouse *klotho* gene, reducing its expression without completely abolishing it, thus leading to a hypomorphic allele^[87]^. Although a complete knockout model of *klotho* exists, most studies use the mutant hypomorph (*klotho*^−/−^), which is accepted as a Klotho-deficient mouse^[86]^. In CKD, Klotho deficiency contributes to FGF23 resistance, exacerbating phosphate retention and promoting vascular calcification^[88]^. Klotho-deficient mice exhibit premature aging with a median lifespan of 61 days^[87,89,90]^. These *klotho*^−/−^ mice have normal development, indicating Klotho’s role in anti-aging rather than development. Our group showed that reducing circulating PAI-1 levels in *klotho*^−/−^ mice rescues the premature aging phenotype^[8]^. We demonstrated that t-PA and u-PA cleave FGF23; thus, excess PAI-1 increases FGF23 levels by inhibiting t-PA and u-PA. Genetic deficiency and pharmacological inhibition of PAI-1 reduced circulating FGF23 levels. These results suggest that reducing PAI-1 levels may benefit renal function and overall aging.

### Nuclear PAI-1

Emerging evidence suggests that PAI-1 may have roles within the nucleus. Gehlot *et al*. reported that PAI-1 localizes in the nucleus of human umbilical vein endothelial cells, using immunofluorescence and nuclear fractionation, and identified putative nuclear export signals^[91]^. Additionally, PAI-1 may translocate to the nucleus in bladder cancer, suggesting a possible role in transcriptional regulation^[92]^. In the context of colon cancer, it has been shown that PAI-1 directly binds to p65 of the NFκB complex and promotes the gene expression of NFκB’s transcriptional profile^[93]^. However, the characterization of a possible role for PAI-1 in the nucleus is still in its infancy and further experiments could elucidate potential DNA binding sites and mechanisms in the context of cardiovascular aging.

### Evolutionary advantage of heterozygous PAI-1 deficiency in humans

Complementing experimental data, human studies of PAI-1 deficiency provide clinical insights into its impact on longevity and cardiovascular health. The evidence described above for PAI-1 as a therapeutic target for aging comes from animal and *in vitro* models. For translation to clinical use, compelling data from a “natural experiment” in a human population demonstrates the beneficial effects of lifelong PAI-1 reduction. In 1881, a male with a *SERPINE1* genetic mutation married into a Swiss Amish community. Over a hundred years later, in 1992, descendants of this individual were identified as harboring a rare loss-of-function mutation in *SERPINE1* (c.699_700dupTA)^[94]^. This mutation consists of a dinucleotide duplication leading to a frameshift in the mRNA and a truncated protein, and heterozygous carriers have –50% of normal circulating PAI-1 levels^[95]^. This geographically isolated Amish community offers a rare real-time example of human evolution, where infants are effectively “randomized” as either carriers or non-carriers of the *SERPINE1* mutation, enabling researchers to study the long-term effects of reduced PAI-1 levels.

We studied 177 members of this community, including 43 carriers of the mutation, and found that heterozygous carriers exhibited significantly longer leukocyte telomere length, lower fasting insulin levels, and protection from diabetes mellitus^[95]^. Further, carriers of the null *SERPINE1* allele demonstrated preserved cardiovascular fitness and increased longevity by a median of 10 years. These findings suggest that the mutation may confer biological advantages in terms of metabolism, fertility, and protection from aging-related morbidities. The broader implications of this study extend beyond cardiovascular health, as age-related multisystem loss of resilience is closely linked to chronic diseases like metabolic disorders, cancer, and Alzheimer’s. Indeed, the Amish genetic profiles highlight PAI-1 as a mechanistic contributor to aging-related morbidity in humans, supporting the notion that its inhibition could serve as a powerful intervention for promoting human longevity.

## CONCLUSION

**Figure 3.**
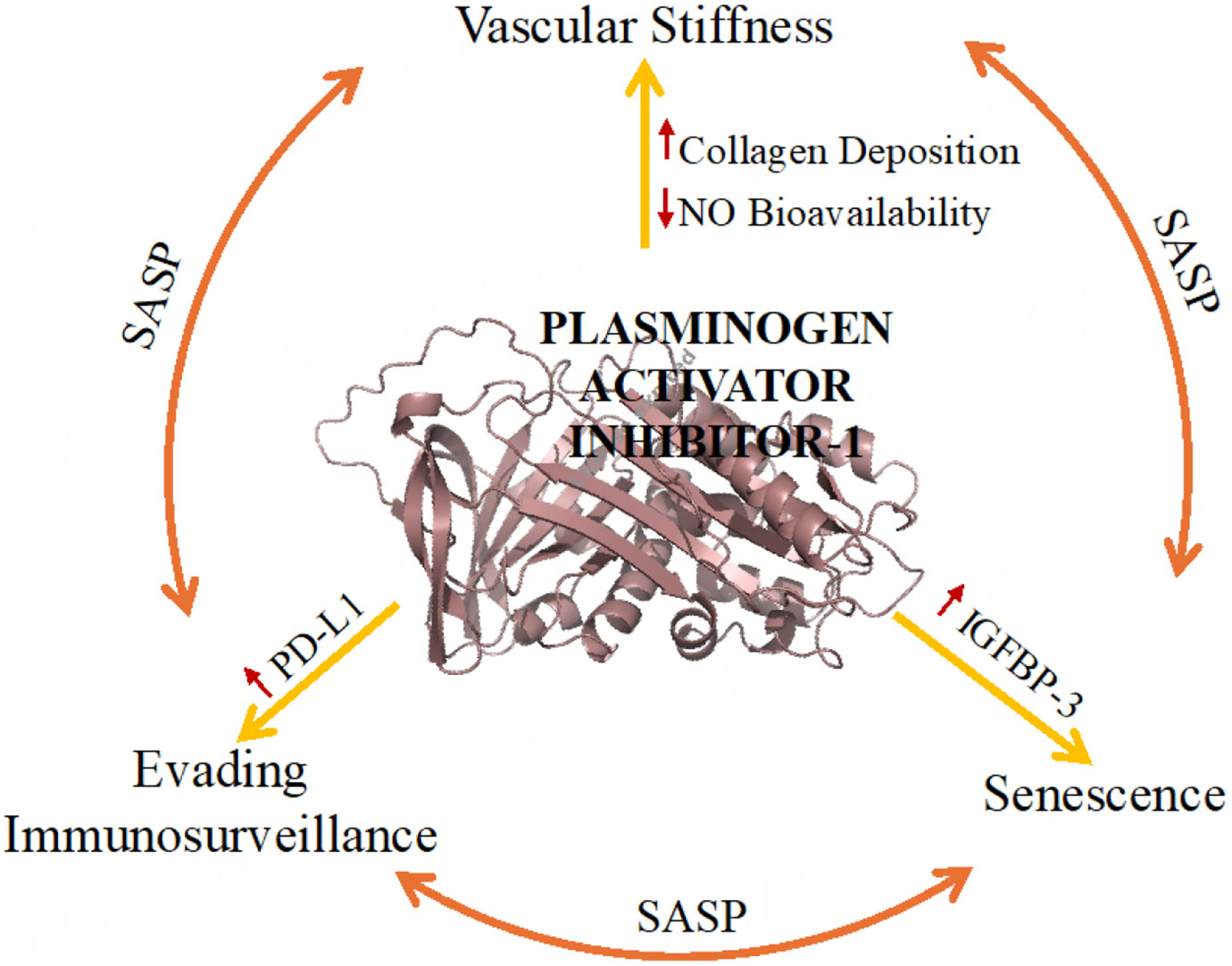
PAI-1 drives cardiovascular aging through interconnected mechanisms: promoting vascular stiffness, amplifying senescence, and enabling immune evasion.

High levels of PAI-1 are intricately associated with a spectrum of negative aging effects that underpin cardiovascular disease, including vascular stiffness, cellular senescence, immune evasion, metabolic dysfunction, and renal impairment. This review has described PAI-1’s role in these processes: it promotes vascular stiffness through collagen deposition and reduced nitric oxide bioavailability, amplifies senescence via senescence-associated secretory phenotype (SASP) factors and IGFBP-3 signaling, and facilitates immune evasion by upregulating PD-L1, thereby linking inflammaging to cardiovascular pathology [Figure 3]. Beyond the vasculature, PAI-1 exacerbates dyslipidemia by enhancing VLDL assembly and impairing fibrinolysis, contributes to renal dysfunction via the FGF23-Klotho axis, and may even influence nuclear transcriptional regulation, as emerging evidence suggests. Conversely, reducing PAI-1 levels through genetic deficiency or pharmacological inhibition mitigates these age-related conditions, as demonstrated in preclinical models and the remarkable longevity observed in humans with heterozygous PAI-1 deficiency.

New technologies promise to deepen our understanding: single-cell RNA sequencing can reveal cell-specific PAI-1 expression patterns in aging vasculature, while advanced imaging techniques like atomic force microscopy offer novel insights into its mechanical effects on ECM. Additionally, CRISPR-based gene editing and high-throughput screening of PAI-1 inhibitors could accelerate the development of targeted therapies, potentially combined with senolytics or senomorphics for synergistic effects.

Given PAI-1’s central role in aging, significant efforts have been made to develop therapeutic strategies aimed at modulating its expression and function, including small molecules and antibody-based inhibitors; many of these approaches have been reviewed extensively elsewhere^[6]^. Several small molecule inhibitors have been developed to target PAI-1 activity, with preclinical and clinical data supporting their therapeutic potential. TM5441 and TM5614, selective PAI-1 inhibitors, have been shown to extend lifespan of *klotho*^−/−^ mice^[89]^ and mitigate senescence-associated fibrosis in the aorta of mice^[38]^. By inhibiting PAI-1’s function, these compounds not only restore fibrinolytic balance but may also influence senescence pathways, positioning them as potential geroprotective agents. Despite the advancements of inhibitors, the pleiotropic functions of PAI-1 necessitate a careful balance between therapeutic benefits and potential off-target effects. Additionally, long-term safety data are needed to establish their suitability for chronic administration. Future directions should focus on refining drug specificity and identifying patient populations most likely to benefit from PAI-1-targeted interventions. Combining PAI-1 inhibitors with other senolytic or senomorphic therapies may offer a comprehensive approach to combating age-related diseases and enhancing human healthspan.
